# Comparison of WHO growth standard and national Indonesian growth reference in determining prevalence and determinants of stunting and underweight in children under five: a cross-sectional study from Musi sub-district

**DOI:** 10.12688/f1000research.23156.4

**Published:** 2021-03-15

**Authors:** Jeannie Flynn, Firas Farisi Alkaff, William Putera Sukmajaya, Sovia Salamah

**Affiliations:** 1 Oeolo Primary Healthcare, Jln. Tetu Neno, Timor Tengah Utara District, East Nusa Tenggara Province, 85662, Indonesia; 2 Department of Pharmacology, Faculty of Medicine, Universitas Airlangga, Jl. Mayjend Prof. Dr. Moestopo No 47, Surabaya, East Java, 60132, Indonesia; 3 Department of Orthopedics and Traumatology, Faculty of Medicine, Brawijaya University, Jl. Jaksa Agung Suprapto No 2, Malang, East Java, 65111, Indonesia; 4 Department of Public Health and Preventive Medicine, Faculty of Medicine, Universitas Airlangga, Jl. Mayjend Prof. Dr. Moestopo No 47, Surabaya, East Java, 60132, Indonesia

**Keywords:** growth chart, Indonesia, risk factors, stunting, underweight

## Abstract

**Background:** Determination of stunting and wasting always uses the WHO growth standard in Indonesia. However, it is believed that Indonesian children are “below” the global standard, thus the WHO standard is not reliable to present the actual prevalence. This study aims to compare the prevalence and determinants of stunting and underweight using WHO growth standard and national Indonesian growth reference.

**Methods: **This was a cross-sectional study carried out in Musi sub-district, East Nusa Tenggara province in July 2019. East Nusa Tenggara province had the highest prevalence of stunting and underweight in Indonesia. The study population were children under five, and total sampling method was used for this study. Length/height-for-age and weight-for-age were plotted using WHO standard and national Indonesian reference. Univariate and multivariate binomial logistic regression were used for statistical analysis.

**Results: **The prevalence of stunting and underweight were higher for the WHO standard than the national reference (53.9% vs 10.7% and 29.17% vs 17.7%; all p < 0.001). After adjusted for confounding factors, when the WHO standard was used, determinants of stunting were maternal mid-upper arm circumference below 23.5cm and maternal height below 150cm; determinants of underweight were intrauterine growth restriction, young maternal age during pregnancy, and multiple parities. When the national reference was used, no determinants was found for stunting; the determinants of underweight were intrauterine growth restriction and maternal education.

**Conclusions:** The WHO standard is not suitable for representing child growth in Musi sub-district. Future studies should be done to re-evaluate the prevalence and determinants of stunting and underweight nationwide using the national Indonesian reference.

## Introduction

According to the data from 2011, the global incidence of stunting, underweight, and wasting were approximately 164.8 million (25.7%), 100.7 million (15.7%), and 51.5 million (8%) among children under five, respectively. Meanwhile, the global deaths attributed to stunting, underweight, and wasting were approximately 1.017 million (14.7%), 999,000 (14.4%), and 875,000 (12.6%) ^1^. Until 2018, stunting and wasting rates remained alarming, although the prevalence was declining. Among continents, Asia has the highest prevalence of stunting (55%) and wasting (68%). Based on country income classification, 65% of all stunted and 73% of all wasted children live in lower- and middle-income countries ^2^. However, in the 2018 report, there is no updated data regarding the prevalence of underweight.

The latest basic health survey in Indonesia in 2018 showed that the prevalence of stunting, underweight, and wasting was 30.8%, 17.7%, and 10.2%, respectively. Among other provinces in Indonesia, East Nusa Tenggara province has the highest prevalence of stunting and underweight, at 42.6% and 29.5%, respectively. Meanwhile, the prevalence of wasting was lower, ranked 8 ^th^ out of 35 provinces ^3^. According to child growth severity classification, the severity of stunting is high and underweight is medium in Indonesia. In East Nusa Tenggara province, the severity of stunting is very high, and the severity of underweight is high ^4^.

However, determination of stunting and wasting always uses the WHO growth standard in Indonesia. It is believed that Indonesian children are “below” the global standard in general, thus the WHO standard is not reliable to present the actual prevalence. Therefore, the national Indonesian growth reference was made using data from National Basic Health Survey ^5^. To this date, no study has been done to scrutinize the difference between WHO growth standard and national Indonesian growth references. This study aims to compare the prevalence and determinants of stunting and underweight using WHO growth standard and Indonesian growth reference. We use the data from one of the sub-districts in East Nusa Tenggara province because this province had the highest prevalence of stunting and underweight among children under five in Indonesia.

## Methods

### Ethical statement

This study followed the principles of the Declaration of Helsinki and was approved by the Department of Health Timor Tengah Utara district (approval number: DINKES.440/995/XI/2019). This study also complies with STROBE guidelines ^6, 7^. All parents gave their written informed consent prior to their children’s inclusion in the study. Information for informed consent was given before the informed consent form was signed. Details that might disclose the identity of the study subjects were omitted from the published data file.

### Study design and population

This study was an observational cross-sectional study conducted in Musi sub-district, one of the sub-districts in East Nusa Tenggara province. Participant recruitment and data collection were conducted in July 2019. Data analysis was conducted in October – December 2019. There were six villages in Musi sub-district. The study population were children aged less than five years old. Total sampling was used for this study. The children and their parents were approached face-to-face by JF during the monthly growth monitoring program in Posyandu (“Pos Pelayanan Terpadu”), a healthcare program by the Indonesian government. Inclusion criteria were children under five who attended the growth monitoring program during the study period, who were born and live with their parents in Musi sub-district, and had maternal and child health books ( *Buku Kesehatan Ibu dan Anak* / KIA) published by the Ministry of Health Republic Indonesia. Children with incomplete KIA was excluded from the determinants analysis.

### Data collection

Both primary and secondary data was used in this study. Primary data for this study consisted of data obtained through interviews with parents, child anthropometry measurements, and maternal height measurements. The interviews took place in the same location as the Posyandu and were conducted by JF using a predetermined questionnaire. The length of the interview was around five minutes. JF is a female general practitioner who worked in primary healthcare in the sub-district where the study took place. She had worked there for seven months when the study was conducted. Interviews with parents was carried out to obtain information regarding village of origin, parents’ highest education, number of parities, delivery method, and sex and age of their children. Anthropometry measurements of maternal height and child length/height were done by healthcare workers from Oeolo Primary Healthcare. Secondary data from KIA was used to obtain data regarding birthweight, gestational age, maternal mid-upper arm circumference, and maternal age during pregnancy.

### Categorization of variables

Underweight and stunting were categorized using WHO growth standard and national Indonesian growth reference for the same sex ^5, 8^. Underweight is defined as weight for age below -2 standard deviations (SD), and severe underweight is defined as weight for age below -3 SD. Stunting is defined as length/height for age below -2 SD, and severe stunting is defined as length/height for age below -3 SD. The cut-off level for maternal mid-upper arm circumference was 23.5 cm, for maternal height was 150 cm, and for children’s birthweight was 2500 g. The cut-off level for maternal mid-upper arm circumference was according to the Indonesian national cut-off ^9^, while for maternal height and children’s birthweight, the cut-off was based on a previous study ^10^. Maternal age during pregnancy was categorized to <20 years old, 20–35 years old, and >35 years old. Gestational age and intrauterine growth were categorized based on Lubchenco charts. It categorizes the gestational age to preterm (<37 weeks), term (37–42 weeks), or postterm (>42 weeks) and the intrauterine growth to small for gestational age (SGA) (<10 ^th^ percentile), appropriate for gestational age (AGA) (10 ^th^ – 90 ^th^ percentile), or large for gestational age (LGA) (>90 ^th^ percentile) ^11^.

### Statistical analysis

Acquired data was analysed using SPSS Statistic for Windows, version 25.0 (IBM Corp., Armonk, N.Y., USA). Data analysis was conducted in two phases. In the first phase, univariate logistic regression was used to identify independent variables that were associated with stunting or underweight. Variables with p < 0.1 were included in the next phase. In second phase, multivariate logistic regression using backward selection was used. Variables with p <0.05 from multivariate analysis were considered as the determinants.

## Results

**Figure 1.**
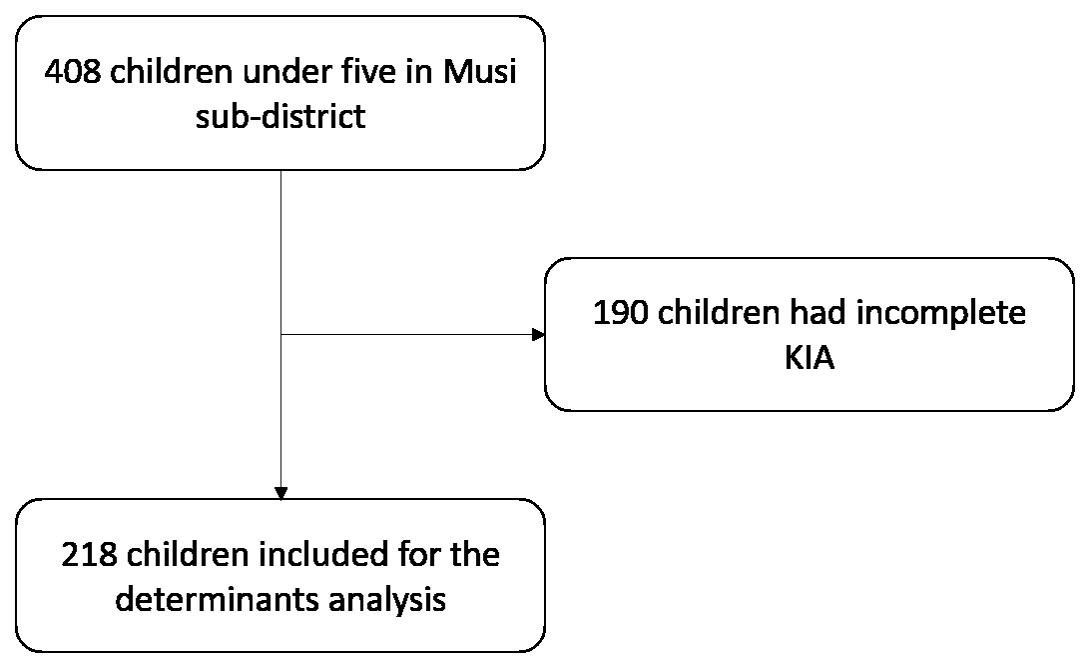
Flow chart of the study population selection for determinants analysis.

There was a total of 408 children under five in Musi sub-district. Based on WHO standard, the prevalence of stunting and underweight were 53.9% and 29.17%, respectively ^12, 13^. Using national reference, the prevalence of stunting and underweight were 10.7% and 17.7%, respectively. There was a significant difference of stunting and underweight between the prevalence from the WHO standard and national reference (both p <0.001). However, there were only 218 children that fulfilled the criteria to be included for the determinants analysis ( Table 1) ( Figure 1).

**Table 1.** Prevalence of stunting and underweight of children aged 0 – 59 months in Musi sub-district.

Variable	Total prevalence (N = 408) n (%)		p-value	Study participants (N = 218) n (%)		p-value
	WHO	National		WHO	National	
Stunting (length/height for age index) Normal (-2 SD and above) Stunted (<-2 SD to ≤-3 SD) Severely stunted (<-3 SD)	188(46.1) 148(36.3) 72(17.6)	364(89.22) 41(10.05) 3(0.73)	< 0.001 *	106(48.6) 75(34.4) 37(17)	200(91.7) 17(7.8) 1(0.5)	< 0.001 *
Underweight (weight for age index) Normal (-2 SD and above) Underweight (<-2 SD to ≤-3 SD) Severely underweight (<-3SD)	289(70.83) 96 (23.53) 23 (5.64)	336(82.3) 57(14) 15(3.7)	< 0.001 ^#^	149(68.3) 55(25.3) 14(6.4)	176(80.7) 33(15.2) 9(4.1)	< 0.001 ^#^

Chi square test was used.

*p-value between stunted (and severely stunted) and normal.

^#^p-value between underweight (and severely underweight) and normal.

p <0.05 was considered statistically significant.

### Sociodemographic characteristics

The prevalence of stunting and underweight among this study population were 51.4% and 31.7% according to WHO standard and 8.3% and 19.3% according to national reference ( Table 1). The number of male and female children were almost equal. More than half of the children were aged between 24 and 59 months old. Majority of the children were born term with a birthweight of more than 2500 g. The education level of both parents was mainly elementary school graduates. Almost half of the mothers had a height of less than 150 cm and more than half of the mothers had a mid-arm circumference of ≤23.5 cm during pregnancy ( Table 2).

**Table 2.** Sociodemographic characteristics among children aged 0–59 months in Musi Sub-district.

Characteristic	Total		WHO		National	
	(N = 218)		Stunted	Underweight	Stunted	Underweight
	n	%	%	%	%	%
Infant						
Child’s sex Male Female	108 110	49.5 50.5	52.8 50	30.6 32.7	7.4 9.1	16.7 21.8
Child’s age group 0–23 months 24–59 months	98 120	45 55	45.9 55.8	29.6 33.3	9.2 7.5	17.3 20.8
Child’s birthweight <2500 g ≥2500 g	35 183	16.1 83.9	65.7 48.6	54.3 27.3	17.1 6.6	40 15.3
Intrauterine growth SGA AGA LGA	38 174 6	17.4 79.8 2.8	65.8 48.9 33.3	57.9 27 0	15.8 6.9 0	44.7 14.4 0
Gestational age Preterm Term Postterm	13 204 1	6 93.6 0.4	61.5 51 0	30.8 31.9 0	15.4 7.8 0	23.1 19.1 0
Parents						
Paternal education No formal education Primary school graduates Secondary school graduates High secondary school graduates University graduates	6 126 32 42 12	2.8 57.8 14.7 19.3 5.5	83.3 54.8 50 45.2 25	50 31.7 28.1 38.1 8.3	33.3 9.5 6.3 4.8 0	50 22.2 15.6 14.3 0
Maternal education No formal education Primary school graduates Secondary school graduates High secondary school graduates University graduates	4 103 35 48 28	1.8 47.2 16.1 22 12.8	75 52.4 65.7 47.9 32.1	75 29.1 40 35.4 17.9	25 8.7 8.6 10.4 0	75 20.4 22.9 18.8 3.6
Maternal MUAC ≤23.5 cm >23.5 cm	125 93	57.3 42.7	43.2 44.1	31.2 32.3	10.4 5.4	22.4 15.1
Maternal height <150 cm ≥150 cm	96 122	44 56	65.6 40.2	40.6 24.6	12.5 4.9	26 13.9
Maternal age during pregnancy <20 years old 20–35 years old >35 years old	20 166 32	9.2 76.1 14.7	70 50.6 43.8	65 25.9 40.6	15 8.4 3.1	40 15.7 25
Number of parities 1 2 3 4 >4	51 65 51 30 21	23.4 29.8 23.4 13.8 9.6	56.9 43.1 60.8 36.7 61.9	35.3 24.6 37.3 16.7 52.4	9.8 7.7 9.8 0 14.3	19.6 18.5 27.5 3.3 23.8

SGA, small for gestational age; AGA, appropriate for gestational age; LGA, large for gestational age; MUAC, mid-upper arm circumference.

### Determinants of stunting according to WHO standard and national reference

Based on WHO standard, univariate logistic regression analysis indicated that children with maternal height below 150 cm (COR = 2.844; 95% CI = 1.632 – 4.956) were more likely to be stunted ( Table 3). In the multivariate logistic regression analysis, other variables with p-value between 0.05 and 0.1 from the univariate analysis (child’s birthweight, child’s intrauterine growth status, maternal mid-upper arm circumference, and number of parities) were included. Multivariate analysis indicated that children with maternal height below 150 cm (AOR = 2.936; 95% CI = 1.672 – 5.154) or maternal mid-upper arm circumference below 23.5 cm (AOR = 1.769; 95% CI = 1.008 – 3.105) were more likely to be stunted ( Table 4).

**Table 3.** Univariate analysis of determinants for stunting among children aged 0–59 months in Musi Sub-district.

Variables	WHO			National		
	COR	95% CI	*p* value	COR	95% CI	*p* value
Infant factors						
Child’s sex Male Female (ref)	1.118 -	[0.657 – 1.902] -	0.682 -	0.8 -	[0.303 – 2.111] -	0.652 -
Child’s age group 0–23 months 24–59 months (ref)	0.672 -	[0.393 – 1.148] -	0.146 -	1.247 -	[0.475 – 3.274] -	0.654 -
Child’s birthweight <2500 g ≥2500 g (ref)	**2.024** -	**[0.951 – 4.310]** -	**0.067** -	**2.948** -	**[1.025 – 8.476]** -	**0.045** -
Intrauterine growth SGA AGA (ref) LGA	**2.014** - 0.524	**[0.967 – 4.192]** - [0.093 – 2.933]	**0.061** - 0.462	**2.531** - 0.0	**[0.885 – 7.239]** - [0]	**0.083** - 0.999
Gestational age Preterm Term (ref) Postterm	1.538 - 0.0	[0.487 – 4.862] - [0]	0.463 - 1.0	2.136 - 0.0	[0.435 – 10.484] - [0]	0.350 - 1.0
Parent factors						
Paternal education No formal education Primary school graduates Secondary school graduates High secondary school graduates (ref) University graduates	6.053 1.465 1.211 - 0.404	[0.65 – 56.365] [0.727 – 2.956] [0.482 – 3.042] - [0.096 – 1.705]	0.114 0.286 0.685 - 0.217	**10** 2.105 1.333 - 0.0	**[1.094 – 91.441]** [0.451 – 9.817] [0.178 – 10.014] - [0]	**0.041** 0.343 0.780 - 0.999
Maternal education No formal education Primary school graduates Secondary school graduates High secondary school graduates (ref) University graduates	3.261 1.198 2.083 - 0.515	[0.316 – 33.614] [0.603 – 2.378] [0.848 – 5.118] - [0.194 – 1.364]	0.321 0.606 0.109 - 0.182	2.867 0.823 0.806 - 0.0	[0.249 – 33.065] [0.260 – 2.604] [0.179 – 3.623] - [0]	0.399 0.741 0.779 - 0.998
Maternal MUAC ≤23.5 cm >23.5 cm (ref)	**1.668** -	**[0.971 – 2.865]** -	**0.064** -	2.043 -	[0.702 – 5.947] -	0.190 -
Maternal height <150 cm ≥150 cm (ref)	**2.844** -	**[1.632 – 4.956]** -	**< 0.001** -	**2.762** -	**[0.997 – 7.655]** -	**0.051** -
Maternal age during pregnancy <20 years old 20–35 years old (ref) >35 years old	2.278 - 0.759	[0.835 – 6.214] - [0.354 – 1.626]	0.108 - 0.479	1.916 - 0.350	[0.500 – 7.346] - [0.044 – 2.762]	0.343 - 0.319
Number of parities 1 (ref) 2 3 4 >4	- 0.574 1.176 **0.439** 1.233	- [0.274 – 1.204] [0.534 – 2.589] **[0.174 – 1.109]** [0.435 – 3.490]	- 0.142 0.687 **0.082** 0.693	- 0.767 1.0 0.0 1.533	- [0.209 – 2.807] [0.271 – 3.689] [0] [0.332 – 7.092]	- 0.688 1.0 0.998 0.584

SGA, small for gestational age; AGA, appropriate for gestational age; LGA, large for gestational age; MUAC, mid-upper arm circumference; COR, crude odds ratio.

**Table 4.** Multivariate analysis of determinants for stunting among children aged 0–59 months in Musi Sub-district.

Variables	WHO			National		
	AOR	95% CI	*p* value	AOR	95% CI	*p* value
Infant factors						
Maternal MUAC ≤23.5 cm >23.5 cm (ref)	1.769 -	[1.008 – 3.105] -	0.047 -			
Maternal height <150 cm ≥150 cm (ref)	2.936 -	[1.672 – 5.154] -	<0.001 -			

AOR, adjusted odds ratio; MUAC, mid-upper arm circumference.

Based on national reference, univariate logistic regression analysis indicated that children with birthweight below 2500 g (COR = 2.948; 95% CI = 1.025 – 8.476) or with a father without formal education (COR = 10; 95% CI = 1.094 – 91.441) were more likely to be stunted ( Table 3). In multivariate logistic regression analysis, other variables with p-value between 0.05 and 0.1 from the univariate analysis (child’s intrauterine growth status and maternal height) were included. No determinant was found in the multivariate analysis ( Table 4).

### Determinants of underweight according to WHO standard and national reference

Based on WHO standard, univariate logistic regression analysis indicated that children with a birthweight below 2500 g (COR = 3.159; 95% CI = 1.507 – 6.622) or intrauterine growth restriction (COR = 3.715; 95% CI = 1.798 – 7.677) were more likely to be underweight. Children with maternal height below 150 cm (COR = 2.098; 95% CI = 1.176 – 3.745) or maternal age under 20 years old during pregnancy (COR = 5.312; 95% CI = 1.989 – 14.186) were also more likely to be underweight ( Table 5). In multivariate logistic regression analysis, other variables with p-value between 0.05 and 0.1 from the univariate analysis (paternal education and number of parities) were included. Multivariate analysis indicated that children with intrauterine growth restriction (AOR = 3.182; 95% CI = 1.450 – 6.980) were more likely to be underweight. Children with maternal age under 20 years old during pregnancy (AOR = 6.252; 95% CI = 1.911 – 20.457) or with mother that had more than four parities (AOR = 4.319; 95% CI = 1.189 – 15.689) were also more likely to be underweight ( Table 6).

**Table 5.** Univariate analysis of determinants for underweight among children aged 0–59 months in Musi Sub-district.

Variables	WHO			National		
	COR	95% CI	*p* value	COR	95% CI	*p* value
Infant factors						
Child’s sex Male Female (ref)	0.904 -	[0.511 – 1.601] -	0.730 -	0.717 -	[0.363 – 1.413] -	0.336 -
Child’s age group 0–23 months 24–59 months (ref)	0.841 -	[0.472 – 1.496] -	0.555 -	0.798 -	[0.403 – 1.580] -	0.517 -
Child’s birthweight <2500 g ≥2500 g (ref)	**3.159** -	**[1.507 – 6.622]** -	**0.002** -	**3.690** -	**[1.680 – 8.107]** -	**0.001** -
Intrauterine growth SGA AGA (ref) LGA	**3.715** - 0.0	**[1.798 – 7.677]** - [0]	**< 0.001** - 0.999	**4.825** - 0.0	**[2.241 – 10.389]** - [0]	**< 0.001** - 0.999
Gestational age Preterm Term (ref) Postterm	0.950 - 0.0	[0.282 – 3.200] - [0]	0.935 - 1.0	1.269 - 0.0	[0.333 – 4.831] - [0]	0.727 - 1.0
Parent factors						
Paternal education No formal education Primary school graduates Secondary school graduates High secondary school graduates (ref) University graduates	1.625 0.756 0.636 - **0.148**	[0.292 – 9.050] [0.365 – 1.564] [0.236 – 1.713] - **[0.017 – 1.255]**	0.579 0.450 0.370 - **0.080**	**6.0** 1.714 1.111 - 0	**[0.973 – 36.986]** [0.656 – 4.481] [0.307 – 4.026] - [0]	**0.054** 0.272 0.873 - 0.999
Maternal education No formal education Primary school graduates Secondary school graduates High secondary school graduates (ref) University graduates	5.474 0.749 1.216 - 0.396	[0.527 – 56.714] [0.362 – 1.553] [0.495 – 2.985] - [0.128 – 1.232]	0.154 0.438 0.670 - 0.110	**13.0** 1.110 1.284 - **0.160**	**[1.207 –139.959]** [0.465 – 2.646] [0.440 – 3.748] - **[0.019 – 1.342]**	**0.034** 0.814 0.647 - **0.091**
Maternal MUAC ≤23.5 cm >23.5 cm (ref)	0.952 -	[0.535 – 1.695] -	0.868 -	1.629 -	[0.803 – 3.303] -	0.176 -
Maternal height <150 cm ≥150 cm (ref)	**2.098** -	**[1.176 – 3.745]** -	**0.012** -	**2.175** -	**[1.095 – 4.318]** -	**0.026** -
Maternal age during pregnancy <20 years old 20–35 years old (ref) >35 years old	**5.312** - **1.957**	**[1.989 – 14.186]** - **[0.892 – 4.296]**	**0.001** - **0.094**	**3.590** - 1.795	**[1.337 – 9.638]** - [0.728 – 4.428]	**0.011** - 0.204
Number of parities 1 (ref) 2 3 4 >4	- 0.599 1.089 **0.367** 2.017	- [0.274 – 1.204] [0.534 – 2.589] **[0.174 – 1.109]** [0.435 – 3.490]	- 0.212 0.837 **0.079** 0.182	- 0.928 0.1551 **0.141** 1.281	- [0.365 – 2.360] [0.615 – 3.913] **[0.017 – 1.166]** [0.379 – 4.336]	- 0.876 0.352 **0.069** 0.690

SGA, small for gestational age; AGA, appropriate for gestational age; LGA, large for gestational age; MUAC, mid-upper arm circumference; COR, crude odds ratio.

**Table 6.** Multivariate analysis of determinants for underweight among children aged 0–59 months in Musi Sub-district.

Variables	WHO			National		
	AOR	95% CI	*p* value	AOR	95% CI	*p* value
Intrauterine growth SGA AGA (ref) LGA	3.182 - 0.0	[1.450 – 6.980] - [0]	0.004 0.999	4.191 - 0.0	[1.820 – 9.649] - [0]	0.001 - 0.999
Maternal education No formal education Primary school graduates Secondary school graduates High secondary school graduates (ref) University graduates				27.341 1.147 1.409 - 0.193	[1.281 - 583.318] [0.412 – 3.188] [0.444 – 4.468] - [0.022 – 1.674]	0.034 0.793 0.561 - 0.136
Maternal age during pregnancy <20 years old 20–35 years old (ref) >35 years old	6.252 - 1.449	[1.911 – 20.467] [0.565 – 3.718]	0.002 - 0.441			
Number of parities 1 (ref) 2 3 4 >4	- 1.283 2.601 0.827 4.319	- [0.480 – 3.430] [0.938 – 7.210] [0.220 – 3.101] [1.189 – 15.689]	- 0.619 0.066 0.778 0.026			

SGA, small for gestational age; AGA, appropriate for gestational age; LGA, large for gestational age; AOR, adjusted odds ratio.

Based on national reference, univariate logistic regression analysis indicated that children with birthweight below 2500 g (COR = 3.690; 95% CI = 1.680 – 8.107) or intrauterine growth restriction (COR = 4.825; 95% CI = 2.241 – 10.389) were more likely to be underweight. Children with mother without formal education (COR = 13.95%; CI = 1.207 – 139,959), with height below 150 cm (COR = 2.175; 95% CI = 1.095 – 4.318), or aged under 20 years old during pregnancy (COR = 3.590; 95% CI = 0.011) were also more likely to be underweight ( Table 5). In multivariate logistic regression analysis, other variables with p-value between 0.05 and 0.1 from the univariate analysis (paternal education and number of parities) were included. Multivariate analysis indicated that children with intrauterine growth restriction (AOR = 4.191; 95% CI = 1.820 – 9.649) were more likely to be underweight. Children with mother without formal education (AOR = 27.341; 95% CI =1.281 – 583,318) were also more likely to be underweight ( Table 6).

## Discussion

In our study, the prevalence of both stunting and underweight were significantly lower when measured using national Indonesian reference compared to when using WHO standard. It has been suggested that overdiagnoses of stunting or underweight are more likely to occur in developing countries ^14^. There are many countries that already proposed their own national growth reference, which are: Korea ^15^, Thailand ^16^, Argentina ^17^, China ^18^, India ^14^, and 18 European countries ^19^. It is argued that the national growth reference of each country is more suitable to reflect the condition in its own population ^16^. However, there were only few published studies that compare the difference between national growth reference and WHO growth standard. A comparison study among Thai children in the first two years of life showed that the prevalence of stunting was higher when using WHO standard in both sexes, but at 24 months the only significant difference was in girls. The prevalence of underweight showed a monotonic increment when using WHO standard, but the Thailand national reference showed a fluctuation ^16^. In Argentina, the prevalence of underweight using WHO standard was 2 times higher than when using their national reference. Meanwhile for stunting, the prevalence when using WHO standard was 1.5 times higher ^17^. In contrary, a comparison study from China showed that the prevalence of stunting and underweight was significantly higher when measured using their national reference ^18^.

The marked difference in measurements using national Indonesian reference and WHO standard probably stems from the difference in methodology during the development of both growth references. The WHO standard was developed using data from six study sites: Brazil, Ghana, India, Norway, Oman, and the USA. The children included in the study were healthy children with suitable sociodemographic conditions for growth. Moreover, all participants agreed to follow the feeding recommendation by WHO ^20^. In contrary, the development of national Indonesian reference did not have any inclusion and exclusion criteria for study participants. It also did not mention the sociodemographic background of the participants or their feeding habits. The study, however, collected data from all 33 provinces of Indonesia to better reflect the growth of Indonesian children. The rationale to develop national Indonesian growth reference was because there is a size difference between Indonesian and US Americans ^5^.

Review article by Beal *et al.* concluded that the determinants of stunting in Indonesia are maternal height and education, child’s sex, premature birth and birth length, exclusive breastfeeding for six months, living area, and household socio-economic status ^21^. In our study, the determinants of stunting according to WHO standard were maternal height less than 150 cm and maternal upper mid-arm circumference <23.5 cm. In contrast, no determinant was found when Indonesian reference was used. It is because the prevalence of stunting according to Indonesian reference was low. The significant difference in stunting prevalence calculated using national Indonesian reference and WHO standard might be because the WHO standard does not represent local growth appropriately due to population differences in height ^19^, and Indonesian people are generally shorter than the rest of the world.

Regarding underweight, the determinants were also different according to the two different references. However, there was one common determinant: intrauterine growth restriction. The difference of underweight prevalence between WHO standard and national Indonesian reference was not as marked as the difference in stunting prevalence; this may explain that there was still one overlapping determinant. The increased odds of stunting, underweight, and wasting in SGA infants are more relevant in low- and middle-income countries ^22^. It is suggested that SGA children are born with lower intrinsic potential for growth due to the persistent effect of growth restriction in utero ^23, 24^.

There were several limitations of this study. Data regarding socioeconomic status could not be obtained due to parents’ unstable monthly income. Data on exclusive breastfeeding cannot be obtained because some of our samples have not yet completed the exclusive breastfeeding period. Data regarding the frequency of diarrhea could not be obtained because this was not well documented in primary healthcare medical records. The sample size of this study was small, particularly the number of stunting children using national Indonesian growth reference was only 18 children. Therefore, it was not adequate to analyze the determinants. These above-mentioned factors should be accounted for in the ensuing studies. Nevertheless, despite all of the limitations, this is the first study that compare the prevalence and determinants of stunting and underweight among Indonesian children under five using WHO growth standard and national Indonesian growth reference.

## Conclusion

The WHO standard is not suitable for representing child growth in Musi sub-district. Future studies should be done to re-evaluate the prevalence and determinants of stunting and underweight nationwide using the national Indonesian reference. An national Indonesian reference for weight-for-height should also be made to re-evaluate the prevalence and determinants of wasting in Indonesia.

## Data availability

### Underlying data

Figshare: Growth standard comparison between WHO and Indonesian Growth Chart-Population Data. https://doi.org/10.6084/m9.figshare.12121938.v5 ^12^

Figshare: Growth standard comparison between WHO and Indonesian Growth Chart-Determinants Data. https://doi.org/10.6084/m9.figshare.12127425.v3 ^13^

### Reporting guidelines

Figshare: STROBE Checklist-Indonesian and WHO Growth Standard Comparison. https://doi.org/10.6084/m9.figshare.12127689.v2 ^6^

Data are available under the terms of the Creative Commons Attribution 4.0 International license (CC-BY 4.0).
