# Supplementary material for: Comparison of WHO growth standard and national Indonesian growth reference in determining prevalence and determinants of stunting and underweight in children under five: a cross-sectional study from Musi sub-district
Source: F1000Res. 2021 Mar 15;9:324. Originally published 2020 May 4. [Version 4] doi: 10.12688/f1000research.23156.4 (PMC7281657; doi:10.12688/f1000research.23156.4)
Supplement: Peer review history

# Peer review: Reviewer response for version 2

**DOI:** 10.5256/f1000research.26741.r63966

**Published:** 2020-06-08

**Authors:** Aroonsri Mongkolchati

**Affiliations:** 1 ASEAN Institute for Health Development, Mahidol University, Nakhon Pathom, Thailand

Even though there was some limitation of analysis due to the small sample size but the author has already discussed this.

Is the work clearly and accurately presented and does it cite the current literature?

Yes

If applicable, is the statistical analysis and its interpretation appropriate?

Yes

Are all the source data underlying the results available to ensure full reproducibility?

Yes

Is the study design appropriate and is the work technically sound?

Yes

Are the conclusions drawn adequately supported by the results?

Partly

Are sufficient details of methods and analysis provided to allow replication by others?

Yes

Reviewer Expertise:

NA

I confirm that I have read this submission and believe that I have an appropriate level of expertise to confirm that it is of an acceptable scientific standard.

# Peer review: Reviewer response for version 2

**DOI:** 10.5256/f1000research.26741.r63965

**Published:** 2020-06-04

**Authors:** Michael Hermanussen

**Affiliations:** 1 University of Kiel, Kiel, Germany

There are just two minor comments:

1. It is not clear according to what criteria the authors rejected 190 children reducing the total number analyzed from formerly 408 to 218 children. The authors may add this information.
2. Please check. The authors write: “The WHO standard was developed using data from five cities in five different countries: United States, Turkey, Norway, Brazil, and India”. This is not quite true. See [ https://www.who.int/mediacentre/news/releases/2006/pr21/en/; or: https://www.who.int/bulletin/volumes/89/4/11-040411/en/]

Is the work clearly and accurately presented and does it cite the current literature?

Yes

If applicable, is the statistical analysis and its interpretation appropriate?

No

Are all the source data underlying the results available to ensure full reproducibility?

No

Is the study design appropriate and is the work technically sound?

Yes

Are the conclusions drawn adequately supported by the results?

Partly

Are sufficient details of methods and analysis provided to allow replication by others?

Yes

Reviewer Expertise:

NA

I confirm that I have read this submission and believe that I have an appropriate level of expertise to confirm that it is of an acceptable scientific standard.

# Response

**Published:** 2020-06-14

**Authors:** Firas Farisi Alkaff

**Affiliations:** Faculty of Medicine Universitas Airlangga, Indonesia

Dear reviewer,

 Thank you for the comments and suggestions for our manuscript.

 1. It is not clear according to what criteria the authors rejected 190 children reducing the total number analyzed from formerly 408 to 218 children. The authors may add this information.

**Response: **We have added the information why we rejected 190 children for the determinants analysis.

 2. The authors write: “The WHO standard was developed using data from five cities in five different countries: United States, Turkey, Norway, Brazil, and India”. This is not quite true.

**Response: **We have checked and revised our manuscript according to the given reference.

# Peer review: Reviewer response for version 1

**DOI:** 10.5256/f1000research.25560.r63061

**Published:** 2020-05-14

**Authors:** Aroonsri Mongkolchati

**Affiliations:** 1 ASEAN Institute for Health Development, Mahidol University, Nakhon Pathom, Thailand

The abstract was unclear of the result of this study, it should be addressed that after the authors control variable or adjust for confounding factors including mother height and socioeconomic factors. What were the factors have been found to be the determinants factors and this lead to the recommendation and conclusion to be more clear.

 The statistic was used for data explanation should be more clear such as OR mean Cluded OR, COR, by bivariate whereas Adjusted OR, AOR, used for multivariate analysis.

 The conclusion should consider one more limitation is that the small sample size of the data particularly on child stunning number on national WHO standard in comparing. As a result, the next research should be more explored.

Is the work clearly and accurately presented and does it cite the current literature?

Yes

If applicable, is the statistical analysis and its interpretation appropriate?

Yes

Are all the source data underlying the results available to ensure full reproducibility?

Yes

Is the study design appropriate and is the work technically sound?

Yes

Are the conclusions drawn adequately supported by the results?

Partly

Are sufficient details of methods and analysis provided to allow replication by others?

Yes

Reviewer Expertise:

Maternal and Child Health

I confirm that I have read this submission and believe that I have an appropriate level of expertise to confirm that it is of an acceptable scientific standard, however I have significant reservations, as outlined above.

# Response

**Published:** 2020-05-19

**Authors:** Firas Farisi Alkaff

**Affiliations:** Faculty of Medicine Universitas Airlangga, Indonesia

Dear reviewer,

 Thank you for the comments and suggestions to our manuscript.

 1. The abstract was unclear of the result of this study, it should be addressed that after the authors control variable or adjust for confounding factors including mother height and socioeconomic factors. What were the factors have been found to be the determinants factors and this lead to the recommendation and conclusion to be more clear.

**Response: **We have edited the abstract section according to your suggestion.

 2. The statistic was used for data explanation should be more clear such as OR mean Cluded OR, COR, by bivariate whereas Adjusted OR, AOR, used for multivariate analysis.

**Response: **We have changed the OR in the bivariate analysis to Crude OR and OR in multivariate analysis to Adjusted OR throughout the manuscript

 3. The conclusion should consider one more limitation is that the small sample size of the data particularly on child stunning number on national WHO standard in comparing. As a result, the next research should be more explored

**Response: **We have added the small sample size particularly in the number of stunted children according to national Indonesian reference as one of the limitations in our study

# Peer review: Reviewer response for version 1

**DOI:** 10.5256/f1000research.25560.r63062

**Published:** 2020-05-05

**Authors:** Michael Hermanussen

**Affiliations:** 1 University of Kiel, Kiel, Germany

The authors aimed to compare Indonesian growth references for height, weight, and BMI with WHO standards. The authors conclude that the WHO standards over-diagnose stunting and underweight in the Musi sub-district.

 This is an important study as it examines whether the globally used WHO growth charts are applicable also for infants and children of the Musi sub-district, Indonesia.

 Yet, the manuscript needs clarification.

 The authors do not clearly distinguish between the terms “standard” and “reference”:

**Growth references **are statistical summaries of anthropometry, conditioned (usually) on age and sex. References describe how children do grow.

**Growth standards **describe how children should grow. (Garza C, de Onis M. Rationale for developing a new international growth reference. Food Nutr Bull 2004;25(1 Suppl):S5-14.). This is an important distinction. The data presented in this study provide a growth “reference” for the children of the Musi sub-district as they describe how these children grow. They do not describe how these children might grow under different conditions.

 The authors use the terms “stunting”, “wasting”, and “underweight”. These terms refer to body size, they do not describe the nutritional situation.

 Underweight is defined as: weight for age < –2 standard deviations (SD) of the WHO Child Growth Standards median. Stunting is defined as height for age < –2 SD of the WHO Child Growth Standards median. Wasting is defined as weight for height < –2 SD of the WHO Child Growth Standards median ^1^). The three terms relate to an internationally used “standard”, suggesting that this standard is also applicable to the Indonesian population. But this has been questioned. Healthy Indonesians are shorter and lighter than healthy white US citizens (who grow similar to what the WHO considers their growth standard). The difference in size between Indonesian and US Americans was the rational to develop national Indonesian growth references (Pulungan et al. 2018).

 In addition, the authors use the term “undernutrition”. Undernutrition refers to food:

 Undernutrition can be defined as "lack of proper nutrition, caused by not having enough food or not eating enough food containing substances necessary for growth and health" ( https://www.lexico.com/en/definition/undernutrition). Chronic undernutrition has an impact on body size as it results in poor growth, but being short (stunting) is not a synonym of mal- or undernutrition (Scheffler C et al. Stunting is not a synonym of malnutrition. Eur J Clin Nutr. 2019 May 29 ^2^). Short people may be short for other than nutritional reasons.

 It is not clear whether the authors wanted to study size (length and weight), or the nutritional situation of the children of Musi. They start the manuscript with the words: “Undernutrition among children under five …”. Later it is written: “There are three indicators to measure nutritional imbalance that lead to undernutrition …” suggesting that the manuscript is on nutrition. But where is the data on food? The authors need to provide information on food, or at least on energy deposits in terms of skinfold thickness, arm circumferences etc., or in the case of mal- or undernutrition, show clinical signs of protein or calorie malnutrition, or signs of micronutrient deficiencies. This is not the case.

 It is known to the reviewer that there is international confusion about the terms stunting and malnutrition. Calculating the portion of stunted children refers to the question of how many children are below height for age < –2 SD of the WHO Child Growth Standards median. This does not necessarily mean that this portion of children is also undernourished.

 The manuscript needs major rethinking and re-evaluating of the measurements of height and weight. It is necessary to describe growth of the children of Musi, and to publish these data. The children of Musi are shorter and lighter than suggested by the WHO standard. But this does NOT mean that these children suffer from food shortage. It rather appears that the WHO standard is not applicable for these children, and thus does NOT indicate malnutrition of this child population. This needs to be stressed. The authors should carefully read some of the recent papers discussing the misinterpretation of stunting as a sign of undernutrition (Hermanussen et al. The impact of social identity and social dominance on the regulation of human growth. A viewpoint. Acta Paediatr. 2019 Aug 16 ^3^).

 The tables show odds ratios. It is much more informative when data are presented as true values. The reader is not so much interested in what proportion of children ranges above or below a certain cut-off, but what are the mean values of height, weight, etc. in this child population.

 Minor comments:

 Correct the term “national standard”. What you mean is: “national reference”.

 Correct the term “gender”. What you mean is probably “sex”.

 Table 4 needs to be shortened, there is no need to show empty boxes. The same applies for table 6

 Discussion: The authors write: “SGA is a result of poor maternal nutrition during pregnancy”. This is not quite true. Ample evidence obtained from European countries during periods of war and post-war starvation illustrates that even when pregnant women are severely undernourished, the newborn infants only suffer from minor decreases in body weight (Keys A, Brozek J, Henschel A, Mickelsen O, Longstreet Taylor H. The biology of human starvation. The University of Minnesota Press. Minneapolis. 1950.).

 See various comments in the text.

Is the work clearly and accurately presented and does it cite the current literature?

Yes

If applicable, is the statistical analysis and its interpretation appropriate?

No

Are all the source data underlying the results available to ensure full reproducibility?

No

Is the study design appropriate and is the work technically sound?

Yes

Are the conclusions drawn adequately supported by the results?

Partly

Are sufficient details of methods and analysis provided to allow replication by others?

Yes

Reviewer Expertise:

child growth, pediatrics, endocrinology

I confirm that I have read this submission and believe that I have an appropriate level of expertise to confirm that it is of an acceptable scientific standard, however I have significant reservations, as outlined above.

## References

[1] : Country Profile Indicators Interpretation Guide. *WHO*.2010; Reference source

[2] : Stunting is not a synonym of malnutrition. *Eur J Clin Nutr*.2020;74(3) : 10.1038/s41430-019-0439-4 377-386 10.1038/s41430-019-0439-4 31142828

[3] : The impact of social identity and social dominance on the regulation of human growth: A viewpoint. *Acta Paediatr*.108(12) : 10.1111/apa.14970 2132-2134 10.1111/apa.14970 31420898

# Response

**Published:** 2020-05-19

**Authors:** Firas Farisi Alkaff

**Affiliations:** Faculty of Medicine Universitas Airlangga, Indonesia

Dear reviewer,

 Thank you for the comments and suggestions to our manuscript.

 1. The authors do not clearly distinguish between the terms “standard” and “reference”

**Response: **We have distinguished the “standard” and “reference” in our revised manuscript. We use the term “reference” for Indonesian growth chart and use the term “standard” for WHO growth chart.

 2. The authors use the terms “stunting”, “wasting”, and “underweight”. These terms refer to body size, they do not describe the nutritional situation.

**Response: **We do not refer stunting and underweight as nutritional status in our revised manuscript.

 3. It is not clear whether the authors wanted to study size (length and weight), or the nutritional situation of the children of Musi.

**Response: **We have revised the manuscript and limited the aim of our study to body size (length and weight) of the Musi children, not about nutritional status because we do not have the data regarding food, energy deposits, or any clinical sign of undernutrition.

 4. It is necessary to describe growth of the children of Musi, and to publish these data.

**Response: **Unfortunately, we do not have the growth record of children in Musi sub-district. The design of our study is cross-sectional, thus we only have one-time height and weight measurement of the Musi children. We have published the measurement data in online repository ( https://doi.org/10.6084/m9.figshare.12121938.v5 for population data and https://doi.org/10.6084/m9.figshare.12127425.v3 for determinants data)

 5. The authors should carefully read some of the recent papers discussing the misinterpretation of stunting as a sign of undernutrition

**Response: **We have read the corresponding references, and we have made sure to not misinterpret stunting as the sign of undernutrition because the aim of our study is to compare the prevalence of body size using WHO growth standard and Indonesian growth reference.

 6. The tables show odds ratios. It is much more informative when data are presented as true values. The reader is not so much interested in what proportion of children ranges above or below a certain cut-off, but what are the mean values of height, weight, etc. in this child population.

**Response: **We would like to give more informative data by presenting the true values of weight and height. However, since the children are not in the same age, we are not sure whether it is appropriate or not.

 7. Correct the term “national standard”. What you mean is: “national reference”.

 Correct the term “gender”. What you mean is probably “sex”.

 Table 4 needs to be shortened, there is no need to show empty boxes. The same applies for table 6

**Response: **We have corrected the term “national standard” to “national reference” and term “gender” to “sex”. We also have shortened the table 4 and 6 by omitting the empty boxes.

 8. Discussion: The authors write: “SGA is a result of poor maternal nutrition during pregnancy”. This is not quite true.

**Response: **We have removed the discussion regarding the relationship between SGA and poor maternal nutrition.

 9. You might better conclude that "the WHO standard are not suitable for representing child growth in Musi sub-district".

**Response: **We have edited our conclusion according to your suggestion.
